# Neelipleona and Symphypleona (Collembola) from a Sampling in the Mesovoid Shallow Substratum of the Sierra de Guadarrama National Park (Madrid and Segovia, Spain): Taxonomy and Biogeography

**DOI:** 10.3390/insects12030266

**Published:** 2021-03-21

**Authors:** Enrique Baquero, Rafael Jordana, Vicente M. Ortuño

**Affiliations:** 1 Department of Environmental Biology, Faculty of Sciences, University of Navarra, University Campus, 31080 Pamplona, Spain; rjordana@unav.es; 2 Grupo de Investigación de Biología del Suelo y de los Ecosistemas Subterráneos, Departamento de Ciencias de la Vida, Facultad de Ciencias, Universidad de Alcalá, 28801 Madrid, Spain; vicente.ortuno@uah.es

**Keywords:** springtails, new species, mesovoid shallow substratum (MSS), subterranean sampling devices (SSD), Iberian Peninsula

## Abstract

**Simple Summary:**

The material for this study was obtained after intensive sampling in the colluvial mesovoid shallow substratum (MSS) of the Sierra de Guadarrama National Park using 33 subterranean sampling devices (SSD). The data were obtained from the first extraction of the traps between May and October of 2015. This paper presents the results for a small part of the total Collembola captured (4.4% of the total for this sampling), namely, the Neelipleona and Symphypleona. Eleven species belonging to seven families were identified, two of which are new species. Based on the results of this study, and others previously published on Collembola of the MSS in the Sierra de Guadarrama National Park, the presence of epigeal and edaphic species is observed, which, in general, are not as abundant as the newly discovered species. The high abundance and extensive presence in the hypogean environment of most of the new species discovered are indicative that the MSS has a unique and distinct Collembola community.

**Abstract:**

*Megalothorax minimus* (Neelidae) and *Sphaeridia pumilis* (Sminthurididae) had already been identified in surface sampling from Sierra de Guadarrama. In Europe, *Sminthurinus gisini* (Katiannidae) seems to be associated with environments at specific altitudes, and has little representation in this sampling. *Pygmarrhopalites custodum* Baquero and Jordana sp. nov. (Arrhopalitidae) coexists with two previously identified surface occurring species of the same family (*P. elegans* and *Arrhopalites caecus*). However, *P. custodum* is more abundant, indicating that it occupies an ecological niche tending to troglophile in the mesovoid shallow substratum (MSS). Moreover, it is also more abundant in the MSS of higher altitude corresponding to the bioclimatic zones cryo-oro-Mediterranean and oro-Mediterranean supra forest. *Allacma cryptica* Baquero and Jordana sp. nov. (Sminthuridae), is another species that had not been previously detected on the surface in the study area. *A. cryptica* is an addition to a genus which has eight described species. *Gisinurus malatestai* (Sminthuridae) appears well represented in the MSS, being a species present very occasionally in the Mediterranean area. Two species of the genera *Sminthurides* (Sminthurididae) and *Fasciosminthurus* (Bourletiellidae) have been found, but they could not have been identified to the species level. Finally, a few specimens of *Dicyrtomina minuta* (Dicyrtomidae), an abundant species on the surface, have been captured.

## 1. Introduction

Until recently, intensive studies on the mesovoid shallow substratum (MSS) were lacking. Environmental conditions in MSS are similar to those of caves, but have a much more intense dependence on the ground and surface features. In karsts, caves and crevices form by either dissolution or fracture. Animals can be adapted and inhabit these systems, or move through the crevices and reach deep dwelling spaces. Gypsum areas offer similar opportunities to its own specific fauna [1].

Deep crevices or large cavities are generally absent in the granitic medium. Rocks broken and dislodged by surface weathering fall down and roll by gravity, coming to a rest along scree slopes or eventually accumulating in ravines and depressions, forming the MSS.

The MSS of the Sierra de Guadarrama (currently a National Park), being close to Madrid and therefore within easy reach of mainstream research institutions (universities, natural history museums) in the country, has been visited over a century by generations of researchers. Surprisingly though, few studies described the inner fauna of the MSS, and the question of whether such fauna was actually surface fauna that migrated inwards to seek refuge during the unfavorable seasons, or cavity-adapted fauna, was unsettled, prompting us to solve it.

We found that the faunal group contributing the greatest number of species and specimens was Collembola. In previous papers [2,3,4] we described the populations of the genus *Orchesella* and of the taxa Poduromorpha and Entomobryomorpha. In all three studies, the existence of a typical fauna of the MSS was demonstrated for this faunal group through the finding of large populations of hitherto unknown species, never found on the surface, alongside a few typically superficial species that always occurred in small numbers.

With the present study, we wanted to confirm whether the same phenomenon occurred for the Symphypleona, a group of Collembola that, with the exception of some species of the Arrhopalitidae family that can live both on the surface and in caves, is quite precisely epiphyllous or litter-dwelling. This would definitely confirm that MSS has its own characteristic fauna that is separate from the surface system.

## 2. Materials and Methods

### 2.1. Site

The sampling was conducted in the Sierra de Guadarrama National Park, located in the eastern half of the Central System (i.e., the Iberian Peninsula). The park covers 33,960 hectares, with a belt of 62,687.26 hectares that functions as a peripheral protection area [5].

**Figure 1:**
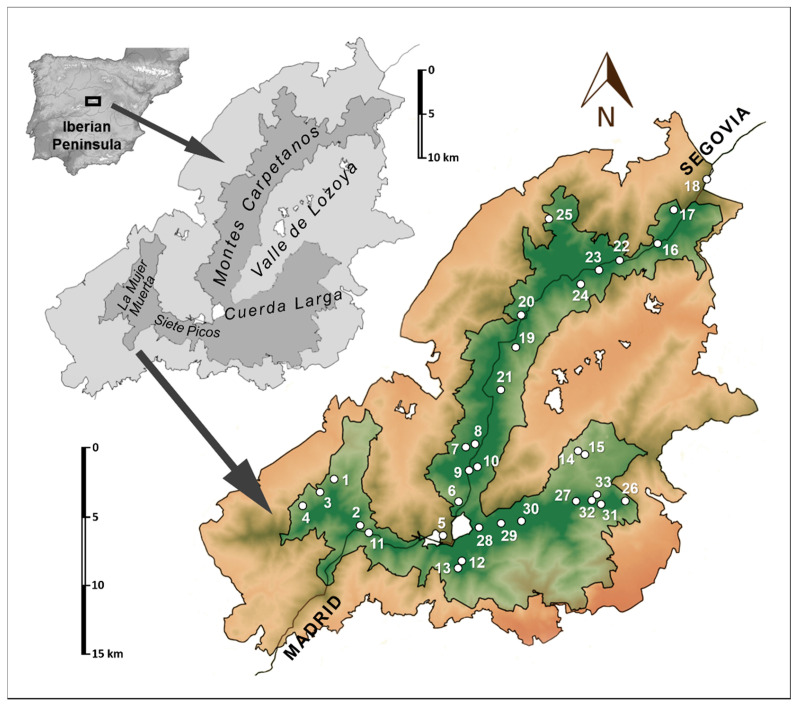
Sampling area in the Sierra de Guadarrama National Park, and locations of the subterranean sampling devices (SSD) used in the study of the Collembola.

The Sierra de Guadarrama mountain range is configured in three axes comprised of the Siete Picos-La Mujer Muerta, Montes Carpetanos, and Cuerda Larga and associated mountainous complex (Figure 1). The dominant rocks are of metamorphic origin of the orthogneiss type [6]. Glacial and periglacial events fragmented this rock typology forming extensive colluvial deposits [7,8] that make up numerous “scree slopes” that allowed the development of the MSS. The studied area is divided into three bioclimatic zones: supra-Mediterranean, oro-Mediterranean, and cryo-oro-Mediterranean [9,10]. The oro-Mediterranean zone is further sub-divided in forest and supra-forest. The most outstanding characteristics of these bioclimatic zones in the Sierra de Guadarrama, and its most conspicuous vegetation, are summarized in [11]. It should also be noted that there is intense snow precipitation in the cryo-oro-Mediterranean and oro-Mediterranean above the scrub supra-forest line.

### 2.2. Methodology

Thirty-three sampling points were established (Figure 1). The sampling was performed mainly using subterranean sampling devices (SSD) that consisted of a PVC cylinder 11 cm in diameter and 1 m in length, with perforations of 8 mm in diameter (with a separation of 15 mm between them) in its lower half, placed in a suitable substrate. A pitfall trap (10 cm in diameter), that fit within the PVC tube, was filled with 1,2-propanediol, and a vial containing cheese, was slid to the bottom of the tube, the top was closed and the entire unit was placed in the soil. Other details that describe the placement of traps and other methodology for capturing the animals have been described in [1]. The authors who performed the sampling included a team that consisted of V. M. Ortuño, E. Ledesma, J. D. Gilgado, A. Jiménez-Valverde, G. Pérez-Suárez, and E. Baquero. Permits to collect samples were obtained from the appropriate authorities (General Directorate of Environment of the Community of Madrid and Territorial Service of the Environment of the Junta de Castilla y León). Traps (Table 1) were placed between 20 May 2015 and 9 July 2015, and the first series of samples were obtained between 17 September 2015 and 6 November 2015.

After the preliminary sorting to separate the Neelipleona and Symphypleona from other Collembola, some representative specimens of each species were selected and mounted in Hoyer’s medium for observation under a compound microscope (phase contrast and Differential Interference Contrast–DIC microscopy). A portion of the specimens were cleared in Nesbitt’s fluid. The remaining samples were stored in 70% ethyl alcohol.

The terminology for *Pygmarrhopalites* Vargovitsh, 2009 [12] used in descriptions follows Fjellberg (1984) [13] for the outer maxillary palp; Nayrolles (1991) [14] for Ant III sensory organ; Bretfeld (1999) [15] for Abd VI; Christiansen (1966) [16] and Christiansen and Bellinger (1998) [17] for *empodium*; and Vargovitsh (2009) [12], for head, body, and legs. The material has been deposited at MZNA—Museum of Zoology at the University of Navarra (Pamplona, Spain).

The abbreviations used are: Abd—abdomen or abdominal segment; Ant—antennal segment or antenna/ae; a.s.l.—above sea level; MSS—mesovoid shallow substratum; SSD—subterranean sampling devices. The chaetae are marked in bold in the text.

## 3. Results

### 3.1. Summary

Neelipleona and Symphypleona accounted for 4.4% of the Collembola captured in the traps (1860 specimens) in the total number of samples used to obtain data for this study (42,745 specimens). Specimens of nine genera and eleven species, belonging to seven families (Neelidae, Sminthurididae, Katiannidae, Arrhopalitidae, Sminthuridae, Bourletiellidae, and Dicyrtomidae) were captured, but with unequal representation. One of the species, belonging to the genus *Pygmarrhopalites* and new to science, was overwhelmingly abundant (1532 specimens; 83%) and had an extensive distribution; a second new species belonging to the genus *Allacma* Börner, 1906 [18], was poorly represented (45 specimens; 2%), and had a more restricted distribution. The other species, with the exception of *Gisinurus malatestai* Dallai, 1970 [19], were almost anecdotal in terms of abundance and distribution.

### 3.2. Taxonomy

Class Collembola Lubbock, 1870 [20]
Neelipleona Massoud, 1971 [21]
Neelidae Folsom, 1896 [22]

#### 3.2.1. *Megalothorax minimus* Willem, 1900

##### Material Studied 

Spain, Sierra de Guadarrama, Segovia, SSD-2, two specimens on a slide (10); SSD-8, two specimens on a slide (10). Madrid, SSD-11, two specimens on a slide (09); SSD-32, one specimen on a slide (01). Ortuño et al. leg [23].

##### Remarks

Present in the Holarctic Region and tropics, and previously referred for Guadarrama by Selga (1971) [24].

##### Ecology

**Figure 2:**
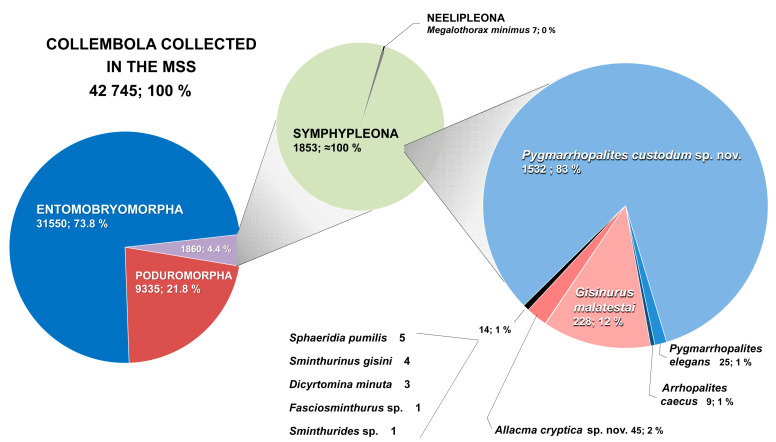
Diversity and abundance of Collembola collected in the MSS of the Sierra de Guadarrama National Park, with special emphasis of the Neelipleona and Symphypleona.

**Figure 3:**
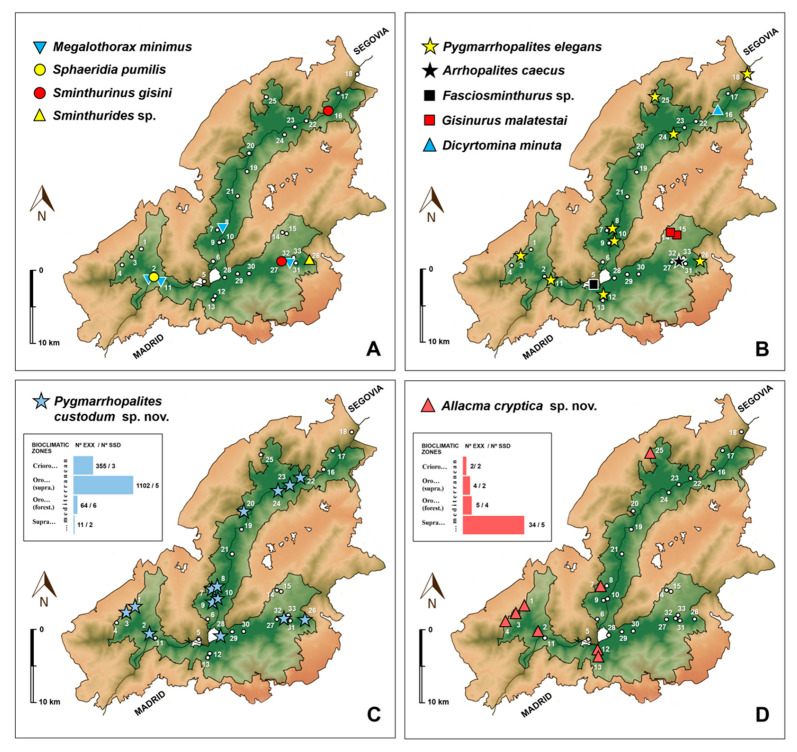
Distribution maps of Neelipleona and Symphypleona species collected in the mesovoid shallow substratum (MSS) of the Sierra de Guadarrama National Park.

Very poorly represented in the MSS of the study area (Figure 2). Observed only in the oro-Mediterranean bioclimatic zone, both in the forest and supra-forest belts. However, it appears to have a wide spatial distribution in the Sierra de Guadarrama National Park, having been detected in the underground of all three mountainous axes (Figure 3A). This species is syntopic with some Symphypleona cited in this work: *Sphaeridia pumilis*, *Allacma cryptica* Baquero and *Jordana* sp. nov., *Pygmarrhopalites elegans*, *Arrhopalites caecus*, and *Pygmarrhopalites custodum* Baquero and *Jordana* sp. nov.

Symphypleona Börner, 1901 [25], sensu Massoud 1971 [21]
Sminthurididae Börner, 1906 [18], sensu Betsch and Massoud 1970 [26]

#### 3.2.2. *Sphaeridia pumilis* (Krausbauer, 1898)

##### Material Studied

Spain, Sierra de Guadarrama, Segovia, SSD-2, four specimens on a slide 03 and one on a slide 11. Ortuño et al. leg [27].

##### Remarks

Type locality: Germany. Present in the Holarctic Region and Australia. Previously cited for the Iberian Peninsula and in Guadarrama [15,28,29].

##### Ecology

Exclusively observed in one of the sampled locations near the Montón de Trigo mountain, part of the Mujer Muerta-Siete Picos mountainous axis (Figure 3A). The MSS is found in a colluvial deposit covered by pine forest (*Pinus sylvestris*) and located at an altitude slightly higher than 1800 m a.s.l., and therefore located in the oro-Mediterranean forest bioclimatic zone. In this MSS, it has been observed that *S. pumilis* is syntopic with *Megalothorax minimus* (Neelipleona), and the Symphypleona, *Pygmarrhopalites custodum* sp. nov. and *Allacma cryptica* Baquero and Jordana sp. nov.

Katiannidae Börner, 1913 [30], sensu Bretfeld 1999 [15]

#### 3.2.3. *Sminthurinus gisini* Gama, 1965

##### Material Studied

Spain, Sierra de Guadarrama, Segovia, SSD-16, one female and one male on a slide (05); Madrid, SSD-27, two juveniles on a slide (03). Ortuño et al. leg [31].

##### Remarks

Originally described from Portugal, and subsequently also found in the Czech Republic and Poland (Tatra Mountains), Slovakia (as *S. carphathicus* Rusek, 1966 [32]) and Austria [33,34,35], sometimes at specific altitudes, up to 1400 m.

##### Ecology

Species poorly represented in the MSS samples (Figure 2). Found in the underground of two mountainous axes (Montes Carpetanos and Cuerda Larga) (Figure 3A), in the oro-Mediterranean forest and cryo-oro-Mediterranean bioclimatic zones. Syntopic with another Symphypleona, *Dicyrtomina minuta*.

Arrhopalitidae Stach, 1956 [36], sensu Bretfeld 1999 [15]

#### 3.2.4. *Arrhopalites caecus* (Tullberg, 1871)

##### Material Studied

Spain, Sierra de Guadarrama, Madrid, SSD-32, one female on a slide (02) and eight in ethyl alcohol. Ortuño et al. leg [37].

##### Remarks

Palearctic species [15] described originally from Sweden, previously cited from Guadarrama [24].

##### Ecology

Found in a very low number of specimens of a single locality (oro-Mediterranean forest bioclimatic zone of the Cuerda Larga mountain axis). In this MSS, it has been observed that *P. caecus* is syntopic with *Megalothorax minimus* (Neelipleona), and *Pygmarrhopalites custodum* sp. nov. (Symphypleona).

#### 3.2.5. *Pygmarrhopalites elegans* (Cassagnau and Delamare-Deboutteville, 1953)

##### Material Studied

Spain, Sierra de Guadarrama, Segovia, SSD-3, two females on a slide (08); SSD-08, one female on a slide (11); SSD-18, three specimens on a slide (02); SSD-25, one specimen on a slide (09) and four in ethyl alcohol; Madrid, SSD-10, one juvenile on a slide (01); SSD-11, one specimen on a slide (10) and seven in ethyl alcohol; SSD-12, one juvenile on a slide (08); SSD-24, two females on a slide (04); SSD-26, two females on a slide (03). All Ortuño et al. leg [38].

##### Remarks

Described originally from the south of the Iberian Peninsula [37], previously cited from Guadarrama [24].

##### Ecology

This species was not very abundant (25 specimens) (Figure 2) in the sample. However, while it has been observed very scarcely in the study area, it is widely distributed across the three mountainous axes of the Sierra de Guadarrama National Park (Figure 3B). This species is present in the subsoil of the three bioclimatic zones, but it appears better represented in the oro-Mediterranean (seven locations out of nine collected), both in the forest and supra-forest belt. In this MSS it has been observed that *P. elegans* is syntopic with *Megalothorax minimus* (Neelipleona), and the Symphypleona *Pygmarrhopalites custodum* sp. nov., and *Allacma cryptica* Baquero and Jordana sp. nov.

#### 3.2.6. *Pygmarrhopalites custodum* Baquero and Jordana sp. nov. (http://zoobank.org/1139329B-EE8F-45D2-A29D-F935DF913018, accessed on 20 March 2021)

**Figure 4:**
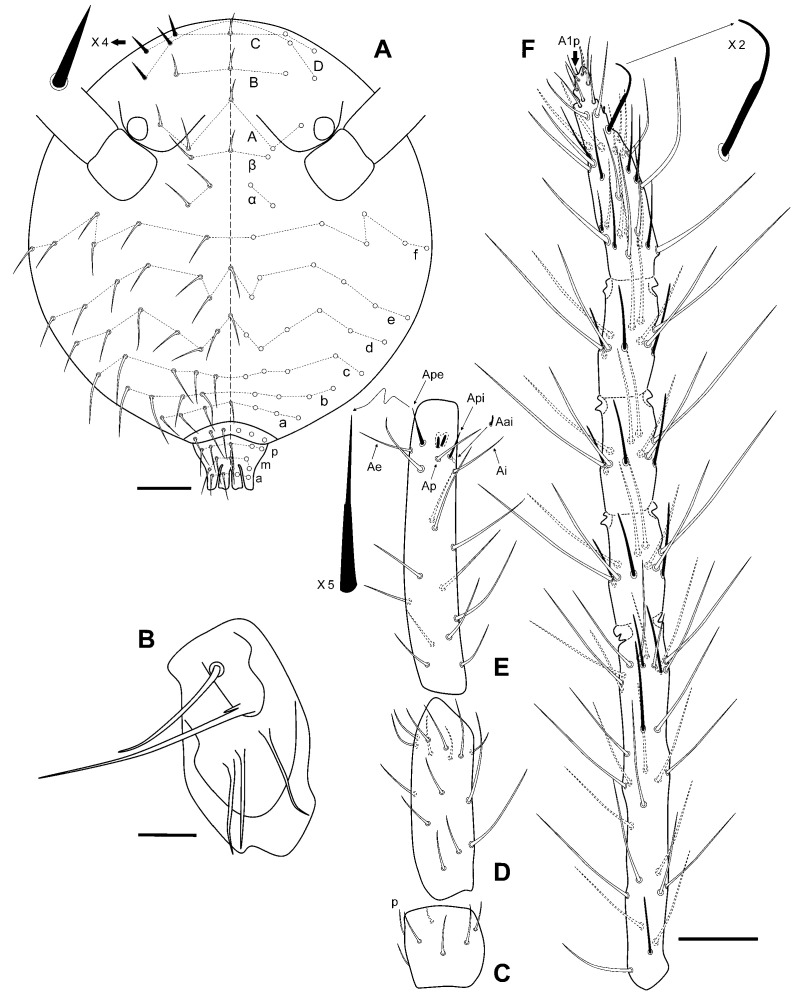
*Pygmarrhopalites custodum* Baquero and Jordana sp. nov.: (**A**) head, dorsal view; (**B**) maxillary palp and sublobal plate; (**C**–**F**) antennal segments I to IV (scale bars: A and C–F, 0.04 mm; B, 0.01 mm).

**Figure 5:**
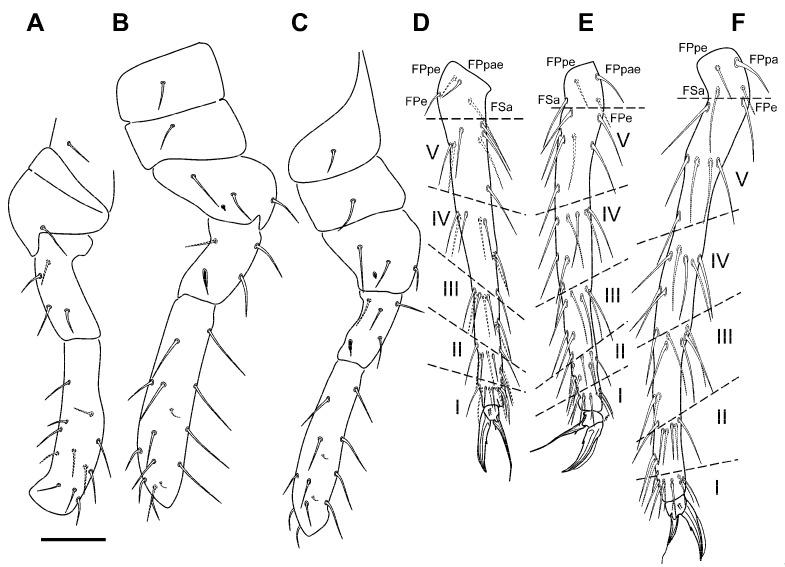
*Pygmarrhopalites custodum* Baquero and Jordana sp. nov.: (**A**–**C**) precoxa 1 to femur of legs 1 to 3 (L1, posterior view; L2–L3, anterior view); (**D**–**F**) tibiotarsus, claw, and empodium of L1 to L3 (all external view) (scale bar: 0.05 mm).

**Figure 6:**
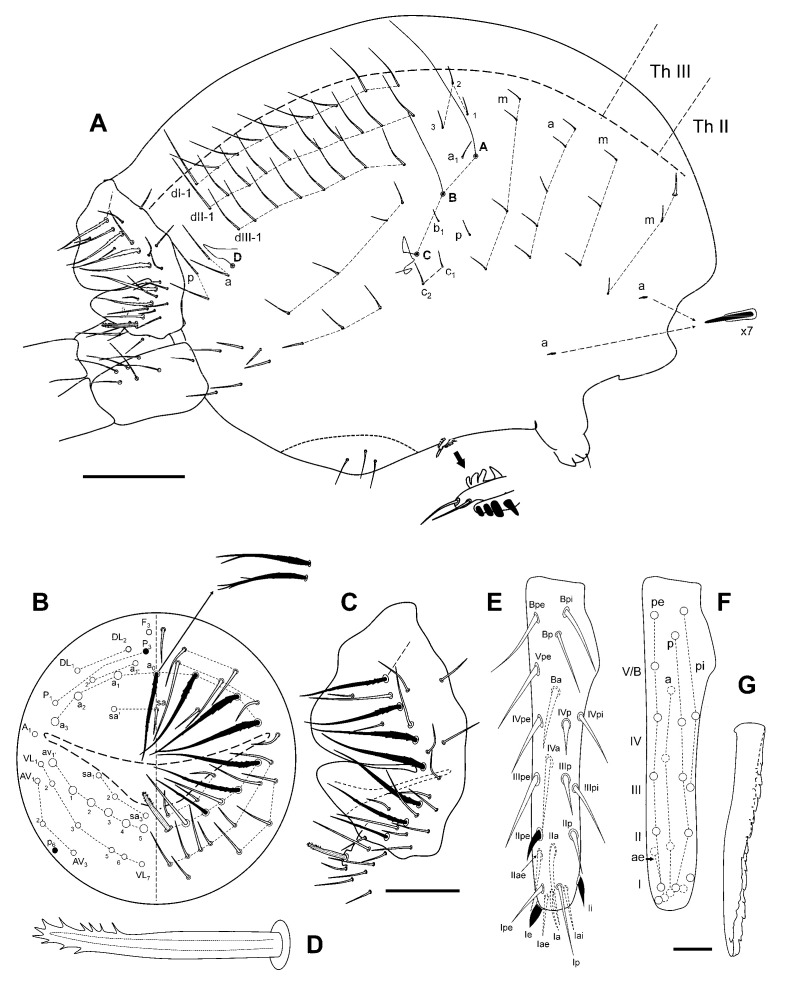
*Pygmarrhopalites custodum* Baquero and Jordana sp. nov.: (**A**) body, lateral view; (**B**) female anal valves schematized; (**C**) female anal valves, lateral view; (**D**) detail of the female anal appendage; (**E**) dens, posterior view; (**F**) dens, schematized; (**G**) mucro (scale bars: A, 0.1 mm; B–C, 0.05 mm; E–G, 0.02 mm).

Figure 4, Figure 5 and Figure 6, Table 2.

##### Type Material

Holotype: female, SSD-20 (slide 03), Montes Carpetanos, Canchal del Cerro de Navahonda (30 T 422698 4533266, 1937 m a.s.l.), Sierra de Guadarrama, Segovia, Spain, 6 October 2015, pitfall SSD (since 24 June 2015), Ortuño et al. leg. Paratypes. Segovia: SSD-7, one female on a slide (10) and 23 specimens in ethyl alcohol; SSD-8, one female and one juvenile on a slide (11) and 41 specimens in ethyl alcohol; SSD-20, five specimens in ethyl alcohol; SSD-22, one female and one juvenile on a slide (09); Madrid: SSD-9, two females on a slide (03) and 146 specimens in ethyl alcohol; SSD-10, two females, one male and one juvenile on a slide (01) and approximately 300 specimens in ethyl alcohol; SSD-23, two females and one juvenile on a slide (05) and 136 specimens in ethyl alcohol; and SSD-24, one female and approximately 725 specimens in ethyl alcohol. Additional material, Segovia: SSD-1, one female on a slide (04) and nine specimens in ethyl alcohol; SSD-2, three females on three slides (01, 11, and 12) and 53 specimens in ethyl alcohol; SSD-3, one female on a slide (08); Madrid: SSD-26, one female on a slide (03); SSD-28, two females on a slide (01) and 66 specimens in ethyl alcohol; and SSD-32, one female on a slide (02).

##### Diagnosis

Eyes 1 + 1. Bothriotricha **ABC** almost aligned. Ant IV with five distinctly separated sub‑segments. Head dorsum with 4 + 4 spine-like chaetae; three unpaired clypeal chaetae. All claws with tunica and inner tooth; *empodia* I–II with corner tooth, III with or without subterminal tooth; all *empodia* with filament, surpassing tips of corresponding claws. *Manubrium* with 7 + 7 posterior chaetae; dens without ventral spines, two outer and one inner spines present; ventral dens formula: 3,2,1,1,0. Abd VI with winged and serrated circumanal chaetae, and anal appendage gutter-like with lateral and terminal fringes (last third approximately).

##### Description

Female. Body length 0.95 mm (head excluded; n = 18; 0.54 to 1.0 mm). Color pattern: pale white background.

Head. Eyes 1 + 1, unpigmented. Clypeal area, row **a**: 3 + axial + 3 chaetae; row **b**: 4 + 4; row **c**: with 5 + 5 chaetae; row **d**: 6 + axial + 6 chaetae; row **e**: 6 + axial + 6 chaetae, row **f**: 6 + 6 chaetae. Inter-antennal area, row **α**: 2 + 2; row **β**: 1 + axial + 1. Lateral chaetae of rows **C** and **D** spine-like (Figure 4A). Chaetotaxy of the mouth region. Labrum: prelabral/labral chaetotaxy: 6/554. 2 + 2 chaetae near the ventral groove. Maxilla: apical chaeta of the maxillary outer lobe (Figure 4B) with a short and thin subparallel branch at the base; sublobal plate with three sublobal hairs.

Antenna (Figure 4C–F) shorter than the body (ratio 0.80) and ratio Ant/head as 1.67 (n = 18); basal sub‑segment of Ant IV 1.23 times longer than Ant III. Ant I with 7 chaetae, **p** as micro chaeta. Ant II with 15 chaetae, two interior ones longer than others. Ant III with 15 chaetae, the two usual sensilla, and three guard sensilla; Ant IV (Figure 4F) with five distinct sub‑segments, with five evident whorls: one at the distal part of basal sub‑segment; one on each of the three intermediate sub‑segments; and another one at the basal part of the terminal sub‑segment. Apical sub‑segment with knobbed subapical organite and short chaeta-like **A1p**; one of the chaetae on this area has a narrowing since terminal half.

Foreleg (Figure 5A,D): precoxae 1, 2, and coxa with 1, 0, 1 chaetae, respectively. Trochanter with three anterior and 1 posterior chaetae. Femur with 12 chaetae, **a4** turned perpendicularly to the longitudinal axis of the segment, **p1** and **p3** thin and short. Tibiotarsus with 43 chaetae: whorl I with 9 chaetae among which **Ja** curved and somewhat thickened, whorls II–V with 8, 8, 8, and 7 chaetae respectively; region F with 3 primary **FP** chaetae (**e**, **ae**, **pe**) and secondary chaeta **FSa**. Pretarsus with 1 anterior and 1 posterior chaetae. Foot complex: claw thin, with reduced tunica, with inner tooth and two pairs of indistinct lateral teeth; *empodium* thin, with corner tooth, and long apical filament surpassing the tip of the claw.

Mid leg (Figure 5B,E): precoxae 1 and 2 with 1, 1 chaetae respectively, precoxal process present, coxa with three chaetae and a microsensillum. Trochanter with three chaetae and a trochanteral organ. Femur with 15 chaetae, **p1** and **p3** very small. Tibiotarsus with 44 chaetae: whorl I with 9 chaetae, whorls II–IV with 8, 8, 8, and 7 chaetae respectively; region F with three **FP** chaetae and **FSa** chaeta. Foot complex: claw wider than foreleg claw, with reduced tunica and inner tooth, and two pairs of small lateral teeth; *empodium* with corner tooth and long apical filament surpassing the tip of the claw.

Hind leg (Figure 5C,F): precoxae 1 and 2 with 1, 1 chaetae respectively, process on precoxa 1 present, coxa with three chaetae and a microsensillum. Trochanter with three chaetae and a trochanteral organ. Femur with 13 chaetae, **p1** and **p3** as micro chaetae. Tibiotarsus with 44: whorl I with 9 chaetae, whorls II–IV with 8, 8, 8, and 7 chaetae respectively; region F with three **FP** chaetae and **FSa** chaeta. Foot complex: claw wider than foreleg claw, with reduced tunica, inner tooth, and two pairs of small lateral teeth; *empodium* with or without a subapical small tooth, and a long apical filament surpassing the tip of the claw.

Large abdomen (Figure 6A): Th II with a single sensillum in row **a** and three chaetae in row **m** with **m_1_** bigger than other. Th III with a sensillum in row **a** and three chaetae in row **m**. Abd row **a** with five chaetae, row **m** with four, and three **p** short chaetae, anterior to bothriotrichal complex. Bothriotrichal complex: **ABC** almost in a linear pattern; bothriotrichum **A** with one posterior accessory short chaeta (a_1_); bothriotrichum **B** with one posterior accessory short chaeta (b_1_); bothriotrichum **C** with two anterior accessory short chaetae (**c_1_** and **c_2_**). Posterior lateral complex with seven, and furca base complex with six chaetae. Posterior dorsal complex with three rows with 9, 10, and 8 long chaetae each. Ventral complex with three chaetae. Fifth abdominal segment: with two chaetae and bothriotrichum **D** in row **a**, and two chaetae in row **p**. Sixth abdominal segment (Figure 6B,C): Abd VI with broadened, winged, and serrated circumanal chaetae (**a_0_**, **a_1_**_–**3**_, **av_1′_** and **AV_1_**; sometimes such chaetae are only winged; in some specimens **a_0_** has its tip simple, doble or four-branched); anal appendage gutter-like with lateral and terminal fringes (last third approximately) (Figure 6D).

Ventral tube with 1 + 1 subapical chaetae.

Tenaculum with two apical chaetae on the *corpus*, three teeth, and a basal process on each ramus.

Furca (Figure 6E,F): *manubrium* with 7 + 7 posterior chaetae. Dens (23 chaetae or spine-like chaetae): anterior side with 3, 2, 1, 1,0 chaetae; posterior side with **Ie** and **IIpe** as massive spines, **Ii** moderately spinous, **IIIpi** and **IVpi** not spine-like. Mucro (Figure 6G): both *lamellae* serrated forming a channel at the end. Dens about 1.4× as long as mucro.

Etymology. The name is derived from the Latin term *custōs* (guard, protector), (masculine name in genitive plural) in tribute and recognition of the people who work in the management and protection of the Sierra de Guadarrama National Park.

Remarks. The species that share with *P. custodum* sp. nov. the presence of the reduced distal formula 021 for the dens (number of ventral–external–internal spine–like chaetae); or one eye + eight spine–like chaetae on head vertex; or **a_0_** chaeta not bifurcated + at least **a_1_** winged and serrated at the base on female anal valves are: *A. antrobius* Yosii, 1954 (Japan) [39], *A. macronyx* Vargovitsh, 2012 (Abkhazia, Western Caucasus) [40], *A. potapovi* Vargovitsh, 2015 (Buryat Republic, Russia) [41], *P. cantavetulae* Jordana, Fadrique and Baquero, 2012 (Teruel, Spain) [42], *P. crepidinis* Jordana and Baquero, 2017 (Almería, Spain) (Jordana et al., 2017) [43], *P. dbari* Vargovitsh, 2017 (Abkhazia, Western Caucasus) [44], *P. dudichi* Loksa and Rubio, 1966 (Hungary) [45], *P. kovali* Vargovitsh, 2017 (Abkhazia, Western Caucasus) [44], *P. kristiani* Vargovitsh, 2005 (Ukraine) [46], *P. nigripes* Park and Kang 2007 (Korea) [47], *P. perezi* Arbea, 2013 (Jaén, Spain) [48], *P. principalis pallida* Linnaniemi, 1912 (Holarctic Region, boreo-alpine area) [49], *P. pseudoprincipalis* Vargovitsh, 2009 (Ukraine) [12], *P. salemensis* Soto-Adames and Taylor, 2013 (Illinois, USA) [50] and *P. zloti* Curcic and Lucic, 1997 (Zlotska Pecina Cave, Serbia) [51]. The species that share the presence of two external and one internal spines on dens, in addition to the new species being described, are: *P. dbari*, *P. dudichi*, *P. crepidinis*, and *P. zloti*; the first two have seven and six sub‑segments on Ant IV; *P. crepidinis* and *P. zloti* have 0 and 13 spine-like chaetae on head vertex respectively. The remaining differences among these species are in Table 2.

Ecology. This is the Symphypleona species dominant in the MSS of the study area. This species was observed in 14 representative localities of the three mountainous axes, and therefore indicative of being widely distributed in the MSS of the Sierra de Guadarrama National Park (Figure 3C). Its abundance was truly remarkable, as they comprised 83% (1532 specimens) of the specimens analyzed in this study (Figure 2). Mostly present in MSS of enclaves at altitudes close to 2000 m a.s.l., or higher, in the oro-Mediterranean supra-forest and cryo-oro-Mediterranean bioclimatic zones; and less abundant at altitudes below 2000 m a.s.l., in the oro-Mediterranean forest and supra-Mediterranean zones (Figure 3C). *Pygmarrhopalites custodum* sp. nov. is syntopic with *Megalothorax minimus* (Neelipleona), and the Symphypleona *Sphaeridia pumilis*, *Pygmarrhopalites elegans*, *Arrhopalites caecus*, *Allacma cryptica* Baquero and Jordana sp. nov., and *Sminthurides* sp.

Sminthuridae Lubbock, 1862 [52] *sensu* Deharveng 2004 [53]

#### 3.2.7. *Allacma cryptica* Baquero and *Jordana* sp. nov. (http://zoobank.org/51419F2B-F400-4AB8-B36B-FE7D1E111711, accessed on 20 March 2021)

**Figure 7:**
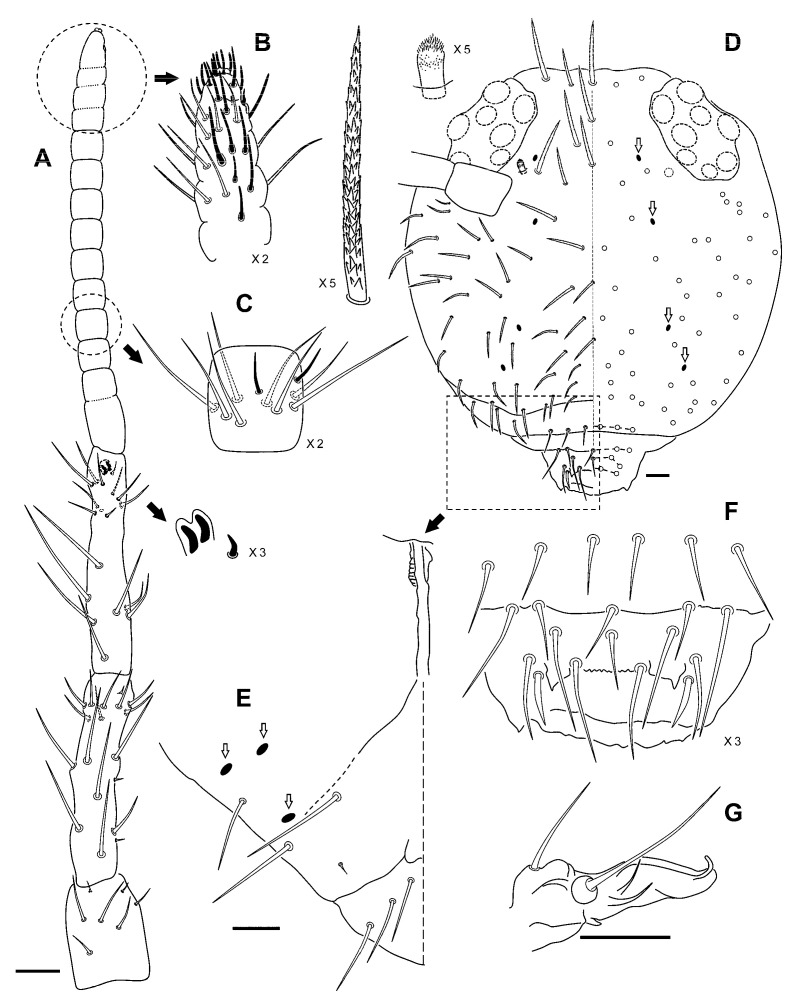
*Allacma cryptica* Baquero and Jordana sp. nov.: (**A**) antenna; (**B**) detail of the antennal tip; (**C**) detail of one of the Ant IV medial sub‑segments (the remaining are similar); (**D**) head, dorsal view, with detail of one of the Mc from vertex and post-antennal special chaetae (oval organs pointed with white arrows); (**E**) head, partial ventral view to show the disposition of the three oval organs (white arrows); (**F**) labrum; (**G**) maxillary palp and sublobal plate (scale bars: all 0.05 mm).

**Figure 8:**
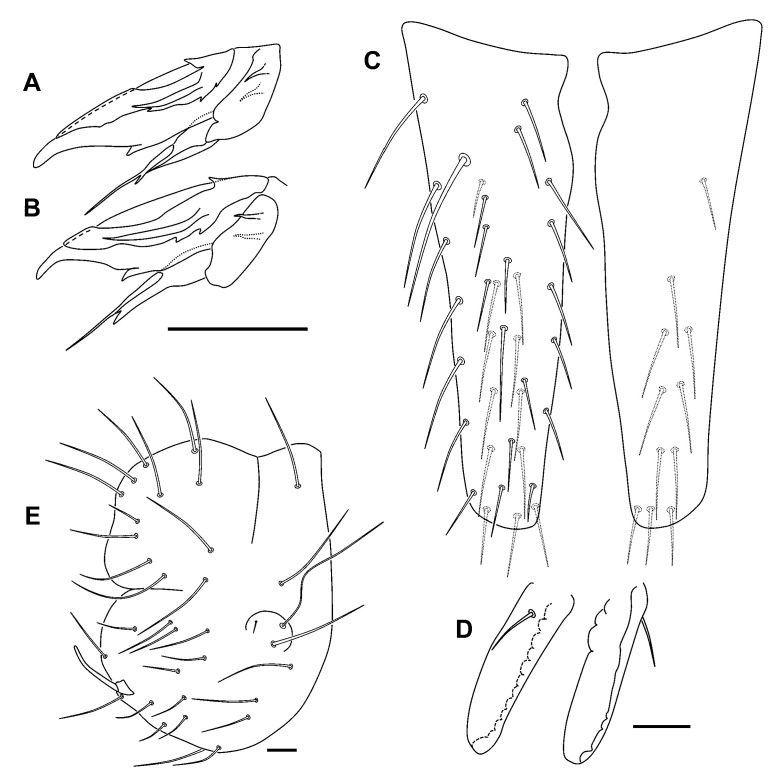
*Allacma cryptica* Baquero and Jordana sp. nov.: (**A**) claw of legs 1 and 2 (are similar), lateral view; (**B)** claw of leg 3; (**C**) dens, posterior view (at right, one of the dens of the Holotype, asymmetric); (**D**) mucro, anterior view at left, posterior view at right; (**E**) female anal valves, lateral view (scale bars: all 0.05 mm).

Figure 7 and Figure 8, Table 3.

##### Type Material

Holotype: female, SSD-12 (slide 07), Siete Picos-La Maliciosa, Canchal Collado del Piornal, north slope of La Maliciosa (30 T 418069 4513856, 2102 m a.s.l.), Sierra de Guadarrama, Madrid, Spain, 22 September 2015, pitfall SSD (since 9 June 2015), Ortuño et al. leg. Paratypes: Segovia, SSD-1, one male and one juvenile on two slides (02 and 07) and 26 specimens in ethyl alcohol; SSD-2, one female and one juvenile on two slides (01 and 11); SSD-3, one female and one juvenile on two slides (04 and 05) and two specimens in ethyl alcohol; SSD-4, one male and one juvenile on a slide (02); SSD-7, four juveniles on two slides (10 and 11); Madrid, SSD-13, one juvenile on a slide (06). Additional material: Segovia, SSD-25, one female and one male on a slide (10 and 11), and one specimen in ethyl alcohol.

##### Diagnosis

Post-antennal chaeta long and slightly pointed, the sculpture of head vertex Mc scale-like and pointed, Ant II and III with 6–5 long chaetae each, 13–15 sub‑segments on Ant IV, two unpaired clypeal chaetae, a conspicuous tooth on the ventral claw, tunica and serrated *pseudonychia* present, *empodium* without an internal tooth, dens anterior with 3, 2, 2, 2, 1…1 chaetae, and anal appendage long, cylindrical, blunt, and without fringes.

##### Description

Female, head length 0.35–0.90 mm; body length 0.73–2.00 mm; antennal length 0.45–1.03; ratio Ant I/II/III/IV as 1/2.53/2.73/4.88 (n = 4); male, head length 0.7 mm; body 1.40–1.45 mm; antennal length 0.75–0.85 mm; ratio Ant I/II/III/IV as 1/3.33/4.00/7.67 (n = 3). Roughly uniform coloration, brownish violet, more intense dorsally in the large abdomen; head with more intense and reddish pigmentation, and antennae and legs, especially the first pair, darker and more violet.

Head. Ant II with 2–3 short ventral chaetae; Ant II and III with 6–5 long chaetae each; Ant IV with 13–15 sub‑segments (Figure 7A–C). Eyes 8 + 8. Head apex with rough chaetae; the remaining chaetae with a very diffuse ciliation; post antennal special chaetae long, pointed or rounded, with fine ciliation (Figure 7D). At least four dorsal and three ventral oval organs present (white arrows in Figure 7D,E). Labral formula 6/5,5,4 (Figure 7F). Maxillary outer lobe bifurcate, with two sublobal hairs (Figure 7G).

Body. Bothriotricha insertion linear or with a slightly obtuse open forward angle; bothriotrichum **D** inserted on small abdomen. Trochanter with a posterior spine-like chaeta. Claws broad, with tunica and serrated *pseudonychia*; inner tooth present on all legs; *empodium* of all legs without an internal tooth, pointed and acuminated, and with a filament not reaching the tip of claw on legs I–II, surpassing the claw on leg III; *pretarsus* with two chaetae (Figure 8A,B). Body with nearly smooth but broad chaetae. Tenaculum with two chaetae on the corpus. *Manubrium* with six dorsal chaetae, in three rows: three distal, two medial, and one proximal. Dens anterior with 3, 2, 2, 2, 1…1 chaetae (the Holotype, asymmetrically, has 3, 2, 2, 2, 2…1); posterior chaetae of normal length, with six internal, five external, eight medial and five proximal (Figure 8C). Mucro with smooth external and lobulated internal edge; mucronal chaeta present (Figure 8D). Small abdomen with normal chaetae, smooth and not broadened at its base; anal appendage long, cylindrical, blunt, and without fringes (Figure 8E).

Etymology. The new species is named *cryptica*, from the Latin *crypticus*, alluding to “hidden”, referring to its subterranean life.

Remarks. The species with which the new species should be compared are *A. fusca* (Linnaeus, 1758) [54], *A. gallica* (Carl, 1899) [55], and *A. koreana* Yosii and Lee, 1963 [56]. A comparison among the above three and the new species, demonstrates differences in the head (post-antennal chaetae shape, vertex macrochaetae sculpture, macrochaetae number and number of sub-segments on the antenna), claws, *empodium*, dens, and anal appendage. The presence of a tooth on the internal claw in all legs allows the differentiation of the new species from *A. fusca* and *A. gallica* (*A. koreana* has it sometimes following Bretfeld 1999 [15]). Another definitive character, to differentiate the new species from *A. fusca* and *A. gallica* is the form of the anal appendage of the female, which in the case of the new species is acuminate while for the other three species it is broad and with a short ciliation. The principal differences among the new species and those previously described are schematized in Table 3.

Ecology. This species was observed in eight localities in the study area; present in the MSS of the three mountainous axes, although not very abundant (45 specimens, with a maximum collection/SSD of 16 specimens) (Figure 3D). However, while these data must be taken with caution given the small number of specimens collected, a trend is observed that it occurs at higher frequency in the MSS at lower altitudes, corresponding to the supra-Mediterranean and oro-Mediterranean forest bioclimatic zones (Figure 3D). *Allacma cryptica* Baquero and Jordana sp. nov. is syntopic with *Megalothorax minimus* (Neelipleona), and the Symphypleona *Sphaeridia pumilis*, *Pygmarrhopalites elegans*, and *Pygmarrhopalites custodum* sp. nov.

#### 3.2.8. *Gisinurus malatestai* Dallai, 1970

##### Material Studied

Spain, Sierra de Guadarrama, Madrid, SSD-14, three females on two slides (04 and 05), and approximately 216 in ethyl alcohol; SSD-15, one female on a slide (06) and eight in ethyl alcohol. All Ortuño et al. leg [19].

##### Remarks

Originally described from Italy, but also found in France (caves) [57], Spain N [58] and Canary Islands [59], and Greece [57].

##### Ecology

Abundant species (228 specimens; 12%; Figure 2) but very localized in the MSS of the Cascada de El Purgatorio area (Figure 3B), located in the Altos de la Morcuera (associated mountainous complex to the Cuerda Larga mountain axis). The two points sampled are located in the supra-Mediterranean bioclimatic zone. No data are available for other Symphypleona or Neelipleona syntopic with *G. malatestai* in the MSS.

#### 3.2.9. *Sminthurides* sp.

##### Material Studied

Spain, Sierra de Guadarrama, Madrid, SSD-26, one juvenile on a slide (03). Ortuño et al. leg.

##### Remarks

It was not possible to determine the identity of the species because a single juvenile was available.

##### Ecology

According to the abundance (Figure 2) and distribution data (Figure 3A), the presence of this species in the MSS of the Sierra de Guadarrama National Park seems anecdotal. This species was found in a locality on the Cuerda Larga mountainous axis, and in the oro-Mediterranean forest bioclimatic zone.

Two other Symphypleona species, *P. elegans*, and *P. custodum* sp. nov., were found in the same locality.

Bourletiellidae Börner, 1913 [30], *sensu* Bretfeld 1994 [60]

#### 3.2.10. *Fasciosminthurus* sp.

##### Material Studied

Spain, Sierra de Guadarrama, Segovia, SSD-5, one juvenile on a slide (05). Ortuño et al. leg.

##### Remarks

It was not possible to determine the identity of the species because a single juvenile was available.

##### Ecology

This species was found in a locality in the confluence of the three mountain axes, and in the oro-Mediterranean forest bioclimatic zone. According to the abundance (Figure 2) and distribution data (Figure 3B), the presence of this species in the MSS of the Sierra de Guadarrama National Park appears anecdotal.

Dicyrtomidae Börner, 1906 [17], sensu Deharveng 2004 [53]

#### 3.2.11. *Dicyrtomina minuta* (Fabricius, 1783)

##### Material Studied

Spain, Sierra de Guadarrama, Segovia, SSD-16, three specimens on a slide (06). Ortuño et al. leg [61].

##### Remarks

A species with wide distribution, both in the Holartic region and in many localities in the southern hemisphere [15]. Until now it had only been identified in the north zone of the Iberian Peninsula [24,62,63] and Portugal S [64]; Selga (1971) [24] cited the subspecies *D. minuta flavosignata* in Madrid.

##### Ecology

The presence of this species in the MSS of the study area is almost anecdotal (Figure 2). It was only observed in the underground of a locality of the Cuerda Larga mountainous axis (Figure 3B), and in the oro-Mediterranean forest bioclimatic zone, being syntopic with another Symphypleona, *S. gisini*.

## 4. General Discussion

Previous intensive Collembola collection activities throughout the 20th century in the Sierra de Guadarrama were conducted in edaphic and epi-edaphic environments, and did not reveal the presence of these species except for *S. pumilis* [29], *A. caecus* [24] and *P. elegans* [24]. Of these species, *S. pumilis* has a surface habit, and the other two have been characterized as troglophiles [65]. Therefore, it came as no surprise that they might have been found in the MSS, which they could use as a refuge. Although cited here for the first time from the Sierra de Guadarrama, *S. gisini* and *D. minuta* are widely-distributed species and thus also likely seeking refuge in the MSS. However, as Figure 2 shows, it does not seem fortuitous that the most abundant species (*P. custodum* sp. nov., *G. malatestai*, *A. crypticae* sp. nov., *P. elegans*, and *A. caecus*) are all either troglobite or troglophilous species. Accounting for 83% of the combined abundance of Symphypleona, *P. custodum* sp. nov. is the dominant species of the group in the MSS of the Sierra de Guadarrama. It is seconded by *G. malatestai*, a very rare species in Europe that has been described as either superficial or troglophilous and found only in very sparse populations, but that in the MSS is rather abundant (223 ex.). We thus suspect that this species belongs to the MSS or caves proper. *Allacma crypticae* sp. nov. is the next most abundant species (45 ex.). The genus had never been cited in the Sierra de Guadarrama, and it also seems a characteristic species of the MSS.

The MSS has more moderate maximum and minimum temperatures than those found in the epigean environment, but this does not mean that the different MSS localities sampled in the study area are comparable. This circumstance is evidenced by the association of *P. custodum* sp. nov. with underground spaces. The number of specimens obtained for *P. custodum* sp. nov. (83% of the total of Symphypleona and Neelipleona collected) allows us to safely conclude that it has a preference in the MSS at higher altitudes (Figure 3C): cryo-oro-Mediterranean and oro-Mediterranean supra-forest (≈95% of specimens) vs. oro-Mediterranean forest and supra-Mediterranean (≈5% of specimens) (Figure 2).

Our data indicate that the MSS has a unique and distinct Collembola fauna, constituting a new biotope for these Hexapoda. Epigean and edaphic species have also been collected in the MSS but, in general, they are less abundant and are less widespread in the underground of the studied area.

This study, comprises a new step toward a more thorough knowledge base and understanding of the MSS biocenosis. It demonstrates that the MSS region has enormous potential to serve as a refuge for surface and subterranean faunal species. Furthermore, it indicates that the management objectives for the MSS should include research of the biodiversity of its unique and distinct natural spaces.

## Figures and Tables

**Table 1:** Location of traps (SSD, subterranean sampling devices).

Mountain Areas of the Sierra de Guadarrama	Code	UTM Coordinates (100 × 100 m)	Altitude (m a. s. l.)	Toponymy/Province	Date Installation of Traps	Date of Trap Recovery	Orientation
Siete Picos—La Mujer Muerta	SSD-1	30 T 4081 45204	1606	Cancho del Río Peces/Segovia	20 May 2015	17 September 2015	North
	SSD-2	30 T 4100 45166	1818	Corrales de la Majada Minguete/Segovia	20 May 2015	17 September 2015	Northeast
	SSD-3	30 T 4068 45192	1622	Umbría de la Mujer Muerta/Segovia	21 May 2015	17 September 2015	North
	SSD-4	30 T 4056 45181	1685	Majada Conejo/Segovia	21 May 2015	17 September 2015	Northwest
	SSD-11	30 T 4108 45161	1876	Cerro Ventoso/Madrid	9 June 2015	17 September 2015	East
Puerto de los Cotos—Puerto de Navacerrada	SSD-5	30 T 4166 45159	1923	Arroyo Seco/Segovia	27 May 2015	22 September 2015	Northwest
Montes Carpetanos	SSD-7	30 T 4185 45229	1994	Majada Hambrienta/Segovia	2 June 2015	17 September 2015	Northwest
	SSD-8	30 T 4190 45231	2071	Majada Aranguez/Segovia	2 June 2015	17 September 2015	Northwest
	SSD-9	30 T 4187 45211	2208	Dos Hermanas/Madrid	3 June 2015	5 October 2015	East
	SSD-10	30 T 4191 45213	2049	Hoya de la Laguna Grande/Madrid	3 June 2015	5 October 2015	East
	SSD-16	30 T 4334 45389	1956	Las Revueltas—Los Horcos/Segovia	23 June 2015	7 October 2015	West
	SSD-18	30 T 4373 45438	1885	Los Loberos/Segovia	23 June 2015	7 October 2015	Northwest
	SSD-20	30 T 4226 45332	1937	Cerro de Navahonda/Segovia	24 June 2015	6 October 2015	Northeast
	SSD-22	30 T 4304 45376	1995	Alto del Puerto/Segovia	24 June 2015	22 September 2015	North
	SSD-23	30 T 4288 45367	2144	Circo del Pico Nevero/Madrid	25 June 2015	6 October 2015	Southeast
	SSD-24	30 T 4274 45357	2042	Peñacabra/Madrid	25 June 2015	22 October 2015	East
	SSD-25	30 T 4249 45407	1731	Arroyo del Charco (La Cepa)/Segovia	2 July 2015	22 October 2015	Northwest
Cuerda Larga and Associated Mountainous complex	SSD-12	30 T 4180 45138	2102	Collado del Piornal/Madrid	9 June 2015	22 September 2015	North
	SSD-13	30 T 4179 45135	2113	Los Almorchones—Las Buitreras/Madrid	10 June 2015	22 September 2015	Southwest
	SSD-14	30 T 4274 45224	1406	El Purgatorio/Madrid	18 June 2015	5 October 2015	West
	SSD-15	30 T 4273 45224	1375	Hueco de los Ángeles/Madrid	18 June 2015	5 October 2015	Northeast
	SSD-26	30 T 4309 45186	1890	La Najarra—Cuatro Calles/Madrid	2 July 2015	30 October 2015	East
	SSD-27	30 T 4270 45185	2101	Bailaderos/Madrid	2 July 2015	30 October 2015	North
	SSD-28	30 T 4193 45164	2156	Collado de Valdemartín/Madrid	3 July 2015	6 November 2015	North
	SSD-32	30 T 4285 45187	1948	Arroyo de La Najarra/Madrid	9 July 2015	22 October 2015	Northeast

**Table 2:** Comparison among the species that share with *P. custodum* Baquero and Jordana sp. nov. the presence of the reduced distal formula 021 for the dens (number of ventral–external–internal spine–like chaetae); or one eye + eight spine–like chaetae on head vertex; or **a_0_** chaeta not bifurcated + at least **a_1_** winged and serrated at the base on female anal valves.

Species/Character	1	2	3	4	5	6	7	8	9	10	11	12	13	14	15	16	17	18	19	20	21	22	23	24	25	26	27	28	29	30	31	32	33	34	D
*A. antrobius*	1	8	U	5	1	1	2	1	1	2	1	1	1	0 *	2	0	5 *	1 *	1 *	1	1 *	1 *	3 *	1 *	1 *	1 *	6 *	0 *	2 *	2 *	2 *	2 *	2 *	2 *	18
*A. macronyx*	1	0 *	0	0 *	0 *	0 *	0 *	0 *	0 *	0 *	0 *	1	0 *	0 *	1–2	0	0 *	0 *	0	0 *	0	0	0 *	0 *	0	0	9 *	0	3	3	3	3	3	1 *	18
*A. potapovi*	1	13 *	1 *	0 *	1	1	2	1	1	2	1	0–1	1	0 *	1	1 *	5 *	1 *	1 *	1	1 *	1 *	3 *	1 *	1 *	1 *	5	0	3	2 *	3	3	3	3 *	16
*P. cantavetulae*	1	0 *	0	5	1	1	2	1	1	2	1	1	1	1	2	0	2	2	0	1	0	0	3 *	2	1 *	1 *	5	0	3	3	3	2 *	2 *	0	6
*P. crepidinis*	1	0 *	0	5	1	1	2	1	1	2	1	1	1	1	2	0	2	2	0	0–1	0	0	0(1)	0(1) *	0	0	5	0	2 *	2 *	2(4) *	2 *	1 *	1 *	8
*P. dbari*	1	11 *	U	7 *	1	1	1 *	1	1	0 *	1	0	0 *	1	2	0	2	2	0	2 *	0	0	1	1 *	0	0	8 *	0	0 *	1 *	1 *	1 *	3 *	0	13
*P. dudichi*	1	8	0	6 *	0 *	1	2	0 *	1	2	0 *	1	1	1	U	0	2	2	0	1	0	0	U	U	U	U	0 *	U	U	U	U	U	U	U	5
*P. kovali*	1	8	0	5	1	1	1 *	1	1	1 *	1	1	1	1	2	0	2	2	0	2 *	0	0	3 *	1 *	1 *	1 *	8 *	0	1 *	1 *	1 *	1 *	1 *	0	13
*P. kristiani*	1	0 *	0	5	0 *	0 *	2	0 *	0 *	1 *	0 *	1	1	1–2	1	0	1 *	2	0	0 *	0	0	0 *	0 *	0	0	U	0	3	3	2 *	2 *	1 *	0	14
*P. nigripes*	2?	9 *	0	7 *	1	1	2	1	1	2	1	1	1	1	2	0	4 *	2	0	1	1 *	1 *	3 *	1 *	1 *	1 *	2 *	0	3	3	3	3	3	3 *	11
*P. perezi*	1	8	0	5	1	1	2	1	1	2	1	1	1	1	2	0	3 *	2	1 *	1	0	0	3 *	1 *	1 *	1 *	1 *	0	3	3	3	2 *	2 *	2 *	10
*P. principalis pallida*	1	8	0	6 *	0 *	1	2	1	1	2	1	1	1	1	2	U	3 *	2	1 *	1	0	0	3 *	1 *	1 *	1 *	2 *	1 *	1 *	1 *	4 *	4 *	1 *	1 *	16
*P. pseudoprincipalis*	1	8	U	5	1	1	2	1	1	2	1	0	1	1	2	U	4 *	2	0	2 *	1 *	1 *	3 *	1 *	1 *	1 *	2 *	0	1 *	1 *	1 *	1 *	1 *	1 *	15
*P. salemensis*	1	0 *	0	5	1	1	2	1	1	2	1	0–1	1	U	2	0	2	2	0	2 *	0	0	3 *	2	1 *	1 *	2 *	0	3	3	3	3	3	1 *	7
*P. zloti*	1	13 *	0	5	0 *	U	2	1	U	0 *	1	U	0 *	1	U	0	2	2	0	1	0	0	1	1 *	0	0	U	U	U	U	U	U	U	U	5
*P. custodum* sp. nov.	1	8	0	5	1	1	2	1	1	2	1	0–1	1	1	1–2	0	2	2	0	1	0	0	1	2	0	0	5	0 or 2	3	3	3	3	3	0	

Legend for the headers of the columns: (**1**) eyes number; (**2**) head, posterior cephalic setae as spine-like total number (both sides); (**3**) antennal III shape: 0, normal; 1, broadened; 2, with papilla; (**4**) antennal IV, sub‑segments number; (**5**) leg I, inner tooth claw: absent = 0; present = 1; 2 = teeth with filament; 3 = paired teeth; (**6**) leg I, corner tooth of the *empodium*: absent = 0; present = 1, 2= paired teeth; (**7**) leg I, apical filament *empodium*: absent = 0; present = 1; longer than claw = 2; (**8**) leg II, inner tooth claw: absent = 0; present = 1; 2 = teeth with filament; 3 = paired teeth; (**9**) leg II, corner tooth of the *empodium*: absent = 0; present = 1; 2= paired teeth; (**10**) leg II, apical filament *empodium*: absent = 0; present = 1; longer than claw = 2; (**11**) leg III, inner tooth claw: absent = 0; present = 1; 2 = teeth with filament; 3 = paired teeth; (**12**) leg III, corner tooth of the *empodium*: absent = 0; present = 1; 2 = paired teeth; (**13**) leg III, apical filament *empodium*: absent = 0; present = 1; (**14**) large abdomen, bothriotrichal pattern: forming an angle close to 90 degrees opened forward = 0; forming a very obtuse angle or a linear pattern = 1; forming an angle close to 90 opened backward = 2; (**15**) tenaculum, number of setae on the *corpus*; (**16**) dens, distal ventral or anterior seta (ve1): normal = 0; strong spine = 1; (**17**) dens, external spine-like setae number; (**18**) Dens, E1 as spine-like: normal = 0; spine-like = 1; strong articulated spine = 2; (**19**) dens, E2 as spine-like: normal = 0; spine-like = 1; strong articulated spine = 2; (**20**) dens, E3 as spine-like: normal = 0; spine-like = 1; strong articulated spine = 2; (**21**) dens, E4 as spine-like: normal = 0; spine-like = 1; strong articulated spine = 2; (**22**) dens, E5 as spine-like: normal = 0; spine-like = 1; strong articulated spine = 2; (**23**) dens, internal spine-like setae number; (**24**) dens, L1 as spine-like: normal = 0; spine-like = 1; strong articulated spine = 2; (**25**) dens, L2 as spine-like: normal = 0; spine-like = 1; strong articulated spine = 2; (**26**) dens, L3 as spine-like: normal = 0; spine-like = 1; strong articulated spine = 2; (**27**) anal appendix shape: 1, flat with apex and edges brush-like; flat and pectinate = 2; flat and smooth = 3; spatulate with apex and edges serrate = 4; gutter-like with fringed apex and edges = 5; gutter-like with cylindrical apex = 6; bifid or trifid and serrate = 7; palmate, and ciliate = 8; tapering or acuminated = 9; (**28**) female anal valves, seta **a_0_** on anal valve shape: simple = 0; bifurcate = 1; tip with fringes = 2 (**29**) same as (28) for **a_1_**; (**30**) same as (28) for **a_2_**; (**31**) same as (28) for **a_3_**; (**32**) same as (28) for **av_1′_**; (**33**) same as (28) for **av_1_**; (**34**) same as (28) for **av_3_**. U, unknown; *, difference for the character with the new species; D, total number of differences between the species and the new species *; ?, a doubtful data.

**Table 3:** Comparison among the species present in the Palearctic area, based on the more useful characters considered in the original descriptions and other papers: *A. fusca* (Holarctic Region), *A. gallica* (described from France; occurrence: Western and Southern Europe, Mediterranean, North Africa) and *A. koreana* (Korea).

Specie/Character	1	2	3	4	5	6	7	8	9	10	11	12	13	14	15	16	17	18	19	20	21	22	23	24	25	D
*A. fusca*	8	1 *	3	5	5	15	1 *	1	0 *	1	1 *	1 *	1 *	1 *	2	6–8	2 *	2	2	2	1	0	1	1 *	3 *	10
*A. gallica*	8	2	3	3 *	5	13	2	0 *	0 *	U	0–1	0–1	1 *	0	U	6–9	2 *	2	2	2	2 *	1 *	1	0	3 *	8
*A. koreana*	8	3 *	U	4	4	12 *	1 *	1	0–1	1	1 *	1 *	1 *	0	3	7 *	2 *	2	2	2	1	0	1	0	4 *	9
*A. cryptica* sp. nov.	8	2	2–3	4–5	4–5	13–15	2	1	1	1	0	0	0	0	1–3	6	3	2	2	2	1	0	1	0	1–2	

Legend for the headers of the columns: (1) eyes number; (2) post antennal chaetae shape: short and rounded, all ciliated = 1; long, pointed or rounded = 2; rounded, but ciliated only at terminal half = 3; (3) ant II, number of ventral chaetae; (4) ant II, number of thick chaetae; (5) ant III, number of thick chaetae; (6) ant IV sub‑segments; (7) posterodorsal gland on large abdomen: flat = 1; prominent = 2; (8) head, surface sculpture of Mc: scale-like = 0; scale-like and pointed = 1; (9) claw, internal teeth number; (10) claw, presence of tunica: absent = 0; present = 1; (11) empodium L1, presence of internal tooth: absent = 0; present = 1; (12) same as (11) for empodium L2; (13) same as (11) for empodium L3; (14) empodium, additional apical teeth number; (15) empodium filament/claw relation: filament shorter than claw = 1; similar = 2; filament longer than claw = 3; (16) manubrium dorsal, chaetae each side; (17) dens, anterior chaetae, row 1 number; (18) dens, anterior chaetae, row 2 number; (19) dens, anterior chaetae, row 3 number; (20) dens, anterior chaetae, row 4 number; (21) dens, anterior chaetae, row 5 number; (22) dens, anterior chaetae, row 6 number; (23) dens, anterior chaetae, row 7 number; (24) dens, outer and posterior chaetae: normal or similar to the rest = 0; long and knobbed = 1; (25) anal appendage tip shape: pointed = 1; blunt = 2; broadened at its total length = 3; spatulated = 4. U, unknown; *, different for the character with the new species; D, total number of differences between the species and the new species.

## Data Availability

Data are available upon request from the authors.
